# Is *Sporothrix chilensis* circulating outside Chile?

**DOI:** 10.1371/journal.pntd.0008151

**Published:** 2020-03-30

**Authors:** Carlos Alberto Tiburcio Valeriano, Reginaldo Gonçalves de Lima-Neto, Cícero Pinheiro Inácio, Vanessa Brito de Souza Rabello, Ertênia Paiva Oliveira, Rosely Maria Zancopé-Oliveira, Rodrigo Almeida-Paes, Rejane Pereira Neves, Manoel Marques Evangelista de Oliveira

**Affiliations:** 1 Programa de Pós-Graduação em Biologia de Fungos, Universidade Federal de Pernambuco, Recife, Pernambuco, Brazil; 2 Departamento de Medicina Tropical, Universidade Federal de Pernambuco, Recife, Pernambuco, Brazil; 3 Instituto Nacional de Infectologia Evandro Chagas, Laboratório de Micologia, Fundação Oswaldo Cruz, Rio de Janeiro, Rio de Janeiro, Brazil; 4 Laboratório de Taxonomia, Bioquímica e Bioprospecção de Fungos, Fundação Oswaldo Cruz, Rio de Janeiro, Rio de Janeiro, Brazil; Faculty of Science, Ain Shams University (ASU), EGYPT

## Abstract

*Sporothrix chilensis* is a mild-pathogenical specie of *Sporothrix pallida* complex, until now, known as restrict to Chile. Herein, we describe the first clinical isolates identified as *S*. *chilensis* in Brazil, preserved in the URM Culture Collection, by polyphasic taxonomy, and their respective antifungal profile of this emergent fungus.

## Author summary

Fungi of the *Sporothrix schenckii* Complex are the causative agents of the sporotrichosis, subcutaneous mycosis that presents a massive global distribution. In this work, we perform a taxonomic review, by a poliphasic approach, of *Sporothrix* sp. isolates stored in the University of Recife Culture Collection (Micoteca URM), previously identified as *Sporothrix schenckii* by classic methods of taxonomic identification. In addition, we perform the method of broth microdilution, aiming to acquire the minimal inhibitory concentration of the standard antifungals (itraconazole and amphotericin B) against these isolates. By sequencing of the partial β-tub-encoding gene, we confirm the occurrence of *S*. *schenckii*, and identified four isolates as the *S*. *pallida* Complex component, *S*. *chilensis*. All the isolates were susceptible to the antifungals, except one, that shows resistance to amphotericin B. Our study demonstrated the first occurrence of *S*. *chilensis* in Brazil. Also, we provide the first data concerned the resistance profile of this specie for the choice antifungals in the sporotrichosis treatment, what will have important implications with this regard in the future.

## Introduction

Sporotrichosis is a subcutaneous fungal infection with worldwide distribution, presenting some endemic areas in tropical and subtropical regions, occurring preferably Latin America [1]. Is caused by the thermodimorphic fungi of the genus *Sporothrix* which are associated, in the environment, with vegetables and soil [2]. Over the last decade, other *Sporothrix* species besides *Sporothrix schenckii*, such as *Sporothrix brasiliensis*, *Sporothrix globosa*, *Sporothrix mexicana*, *Sporothrix luriei*, *Sporothrix pallida* and *Sporothrix chilensis* have been reported as agents of sporotrichosis [3–10]. This complex of fungi is characterized by major differences in routes of transmission, host tropism, virulence, and antifungal susceptibility [11]

*S*. *chilensis* is a recently described *Sporothrix* species in one clinical case, where the fungus was isolated from a patient with onychomycosis in Chile [10]. Based on this exclusive *S*. *chilensis* human clinical case described up to now, a question emerges about the occurrence of other cases of sporotrichosis associated with this fungus in other countries where sporotrichosis occurs, for instance, Brazil, an important endemic country of this infection with a hyperendemic area of zoonotic sporotrichosis described over the last 20 years [12].

Identification of *Sporothrix* pathogenic species must be performed according to polyphasic criteria. An identification key for the *Sporothrix* pathogenic species has been proposed including analysis of conidial morphology, growth rates at 30 and 37°C, and assimilation of raffinose and sucrose as carbon sources [3]. However, identification based solely on these features is often inconclusive due to phenotypic variability within these species. Therefore, molecular tests are necessary for confirmation [13].

Most studies about the pathogenic species of *Sporothrix* in Brazil are performed in areas with better economic conditions, such as the South and Southeast regions of the country. Furthermore, sporotrichosis is not mandatory notification and the little information on the species distribution and incidence of this disease guide the researchers to access culture collections to understand epidemic that occurring in the Northeast [12]. In the present study, we re-examined, by polyphasic taxonomy, the sub-set collection of *Sporothrix* species of an important Brazilian culture collection hosted by the Department of Mycology of the Federal University of Pernambuco, Northeast Brazil. Isolates from sporotrichosis patients, which were previously classified as *S*. *schenckii sensu lato* (s.l.) based on morphological features, were analyzed by phenotypic and genotypic tests in an attempt to identify the species present in this poorly studied area in the field of sporotrichosis. The susceptibility profile of entire sub-set isolates beforehand identified as *S*. *schenckii sensu stricto* were also evaluated.

## Materials and Methods

### Polyphasic taxonomy

#### Strains

Eleven isolates from the Fungal Culture Collection of the Federal University of Pernambuco, University Recife Mycology (Table 1), previously obtained from patients with confirmed sporotrichosis, between the years 1955 and 2005 in the states of Pernambuco and São Paulo. These isolates were previously identified as *S*. *schenckii* at the moment of preservation, according to their morphological features. The type strains of *S*. *brasiliensis* CBS 120339 (formerly IPEC16490) and *S*. *schenckii* ATCC 32286 were included in this study also. To re-isolate the strains and to obtain pure cultures in the mycelial phase, they were cultured on Sabouraud Dextrose Agar (SDA) slants at 30°C for seven days. Thermoconversion to the yeast-like form was carried out in all isolates at 37°C on Brain Heart Infusion Agar (BHI) for five days.

**Table 1:** Molecular screening by sequencing of the partial β-tub-encoding gene of *Sporothrix* species complex deposited in URM Culture Collection and type strain of *S*. *brasiliensis*.

ISOLATE	SOURCE	MOLECULAR IDENTIFICATION	GENBANK ACESSING NUMBER
**URM434**	Clinical, Pernambuco, Brazil	*S*.*chilensis*	MK478792
**URM435**	Clinical, Pernambuco, Brazil	*S*.*chilensis*	MK478793
**URM2439**	Clinical, Pernambuco, Brazil	*S*.*chilensis*	MK478794
**URM2865**	Clinical, São Paulo, Brazil	*S*.*chilensis*	MK478795
**URM1013**	Clinical, Pernambuco, Brazil	*S*.*schenckii*	MK478785
**URM3686**	Clinical, Pernambuco, Brazil	*S*.*schenckii*	MK478786
**URM4252**	Clinical, Pernambuco, Brazil	*S*.*schenckii*	MK478787
**URM4291**	Clinical, Pernambuco, Brazil	*S*.*schenckii*	MK478788
**URM4861**	Clinical, Pernambuco, Brazil	*S*.*schenckii*	MK478789
**URM5111**	Clinical, Pernambuco, Brazil	*S*.*schenckii*	MK478790
**URM4080**	Clinical, Pernambuco, Brazil	*S*.*schenckii*	MK478791
**CBS120339**	Clinical, Rio de Janeiro, Brazil	*S*.*brasiliensis*	AM 116946

### Molecular screening of isolates

After obtaining pure cultures molecular screening was performed with genomic deoxyribonucleic acid (DNA) extracted from the mycelial phase of the eleven *Sporothrix* isolates by the chloroform/isoamyl alcohol method [13] and screening of at species level by sequencing of the partial β-tub-encoding gene [3]. Automated sequencing was done using the Sequencing Platform at Fundação Oswaldo Cruz—PDTIS/FIOCRUZ, Brazil [14]. Sequences from both DNA strands were generated, edited with the Sequencher ver. 4.6 software package (Genes Codes Corporation, USA), and aligned by means of the Mega version 4.0.2 software. The sequences of our strains were compared by BLAST (Basic Local Alignment Search Tool- NIH) with sequences available from NCBi GenBank (*S*. *schenckii* AM 116911.1/ S. *brasiliensis* AM 116946.1/ *S*. *globosa* AM 116966.1/ *S*. *luriei* AM 747289.1/ *S*. *pallida* EF 139108.1/ *S*. *mexicana* AM498344.1/ *S*. *chilensis* KP 711814.1). All phylogenetic analyses were performed based on method previously described by Tamura and coworkers [15], using MEGA vers. 4.0 (http://www.megasoftware.net/), and the phylogenetic relationships among isolates were evaluated from tree topologies by Maximum Parsimony (MP) algorithm [16] applying the Bootstrap test [17] with 1,000 replicates. Sequences were deposited in GenBank database under accession numbers MK478785 to MK478795 (Table 1).

#### Phenotypic characterization of rare *Sporothrix* species

After molecular screening, four isolates previously characterized by phenotypic methods as *S*. *schenckii sensu stricto* were sub cultured on Potato Dextrose Agar (PDA) plates, and Corn Meal Agar (CMA; BBL Becton, Dickinson and Company/Sparks, MD 21152 USA) slants, and incubated at 30°C in the dark in order to study macroscopic features and sporulation [3]. The microscopic features were determined primarily on CMA slide cultures after 10 to 12 days of incubation at 30°C, and the diameter of the colonies on PDA were measured in triplicate after 21 days according to the previously described protocol [3]. To check growth at 37°C, the strains were grown on PDA and incubated at 37°C for three weeks. Conversion to the yeast form was assessed on Brain Heart Infusion (BHI) Agar (BBL Becton, Dickinson and Company/Sparks, MD 21152 USA) slants after seven days of incubation at 35.5°C. Carbohydrate assimilation tests were performed using freshly prepared Yeast Nitrogen Base (YNB) medium [Difco Becton, Dickinson and Company/Sparks, MD 21152 USA] supplemented with one of three different carbohydrates (dextrose, sucrose, or raffinose) using the technique previously described [3, 13]. Cultures on YNB supplemented with dextrose were used as positive control for growth and YNB without carbohydrates was used as a negative control. Carbohydrate assimilation tests were performed in triplicate runs in different days, under similar laboratorial conditions. The identification, at species level, based on the phenotypic features of the isolates included in this study was evaluated as suggested by Marimon and collaborators [3].

### *In vitro* antifungal susceptibility of *Sporothrix* species determined by molecular screening and reference strains

Reference microdilution trays, containing serial drug dilutions were prepared following the CLSI M38-A2 guidelines [18]. The standard RPMI 1640 medium (Sigma Chemical Co., St., Louis, MO) was buffered to pH 7.0 with 0.165 M of morpholinopropanesulphonic acid (MOPS; Sigma, Brazil).

In order to obtain viable nongerminated conidia (0.4–5 x 10^4^ conidia.mL^-1^), each strain was cultured on a tube containing 20 mL of Potato-Dextrose Agar (PDA; Difco) at 35°C for seven days. After that, conidial suspensions were prepared in sterile physiological solution (0.85%) and adjusted by the spectrophotometer at 530 nm with 80 to 82% of transmitance and 0.09 to 0.013 optical density. *Candida parapsilosis* ATCC 22019 was used as a reference strain. Itraconazole (ITC; Janssen Pharmaceutica, Beerse, Belgium) was evaluated because it is the first choice for the treatment of sporotrichosis [19]. In addition, amphotericin B (AmpB; Bristol-Myers Squibb, Princeton, USA) and terbinafine (TRB; Bristol-Myers Squibb, Princeton, USA) was evaluated. AmpB is indicated in cases of severe disease, and terbinafine is a therapeutic option to sporotrichosis patients that cannot use conventional treatment, such as pregnant women and allergic to the azoles [19–20]. Antifungal drugs were dissolved in dimethylsulfoxide (DMSO) with stock solutions of amphotericin B and itraconazole concentrations of 1.600 μg/mL tested in concentrations ranging from 0.03125 to 16.0 μg/mL, and stock solutions of terbinafine of 6.400 μg/mL tested in concentrations ranging from 0,125 to 64 μg/mL. The inoculated microplates were incubated at 35°C in a non-CO_2_ incubator and were visually examined after 48h. Minimum inhibitory concentrations (MICs) corresponded to the lowest drug dilution that completely inhibited fungal growth as compared to the untreated inoculum. The isolates were tested in the filamentous phase and all tests were performed in triplicate.

## Results

### Isolation and molecular screening of isolates preserved in fungal collection

**Fig 1:**
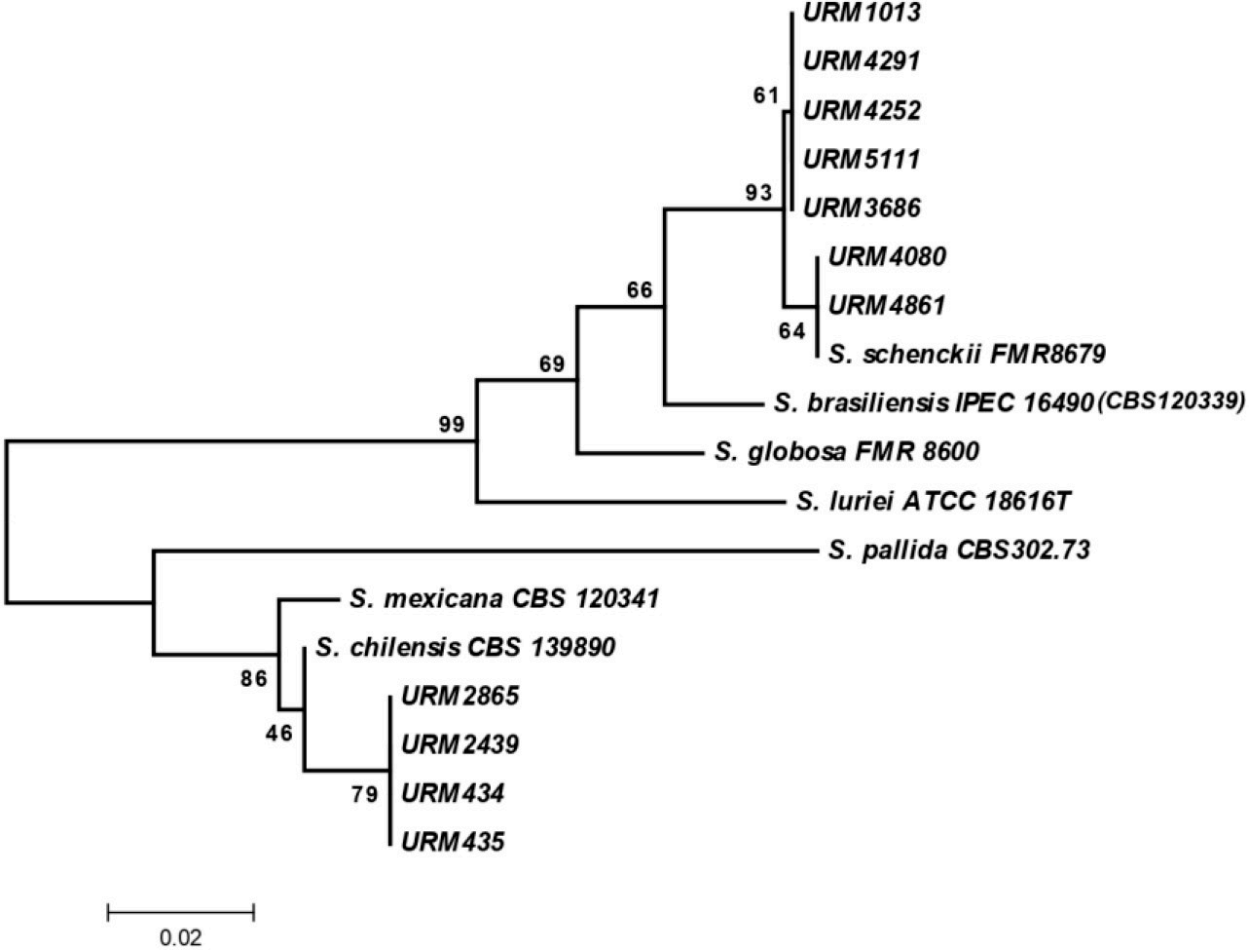
Evolutionary relationships of 18 taxa. The evolutionary history was inferred using the Maximum Parsimony method. Tree #1 out of 107 most parsimonious trees (length = 53) is shown. The consistency index is 0.905660 (0.871795), the retention index is 0.962963 (0.962963), and the composite index is 0.872117 (0.839506) for all sites and parsimony-informative sites (in parentheses). The MP tree was obtained using the Close-Neighbor-Interchange algorithm with search level 2 in which the initial trees were obtained with the random addition of sequences (10 replicates). The tree is drawn to scale, with branch lengths calculated using the average pathway method and are in the units of the number of changes over the whole sequence. The codon positions included were 1st+2nd+3rd+Noncoding. All positions containing gaps and missing data were eliminated from the dataset (Complete Deletion option). There were a total of 175 positions in the final dataset, out of which 24 were parsimony informative. Phylogenetic analyses were conducted in MEGA4.

All isolates were recovered from the URM Culture Collection and pure cultures with macroscopically characteristic of *Sporothrix* species were found in SDA slants at 30°C. After seven days of growth, the initial colonies of these isolates were glabrous, white and, after a few days, a dark pigment was developed in sectors of the mycelia. Microscopic observation at this time revealed the presence of simple, ovate hyaline and thin-walled conidia in sympodial conidiophores, and a second brown, thick-walled dark conidia along the undifferentiated hyphae. At 37°C on Brain Heart Infusion Agar (BHI), all isolates showed growth of moist, glabrous, yeast-like colonies. Microscopically, these isolates presented small, globose or obvoidal, hyaline budding yeast cells. The macroscopic morphologies of the *Sporothrix* spp. studied, as well as the *S*. *brasiliensis* type strain CBS 120339 were similar. Phenotypic characteristics allowed the classification of these fungi as *Sporothrix* and the molecular screening these were available by molecular sequencing data of the partial β-tub-encoding gene with sequences deposited in GenBank as summarized in Table 1. In this molecular screening seven isolates were identified as *S*. *schenckii sensu stricto* and four isolates were confirmed to be members of the *S*. *pallida* complex (Fig 1). GenBank search revealed that four isolates available in this study showed a 99% match with the β-tub sequences of *S*. *chilensis* (i.e., GenBank accession number KP711814.1) and seven isolates showed a 99% match with the β-tub sequences of *S*. *schenckii* (i.e., GenBank accession number AM116922). Type strain of *S*. *brasiliensis* CBS 120339 (IPEC16490) was used as control strain in identification by polyphasic taxonomy since that had already been well studied and presented the same results as previously described [3, 13].

### Phenotypic characterization

**Fig 2:**
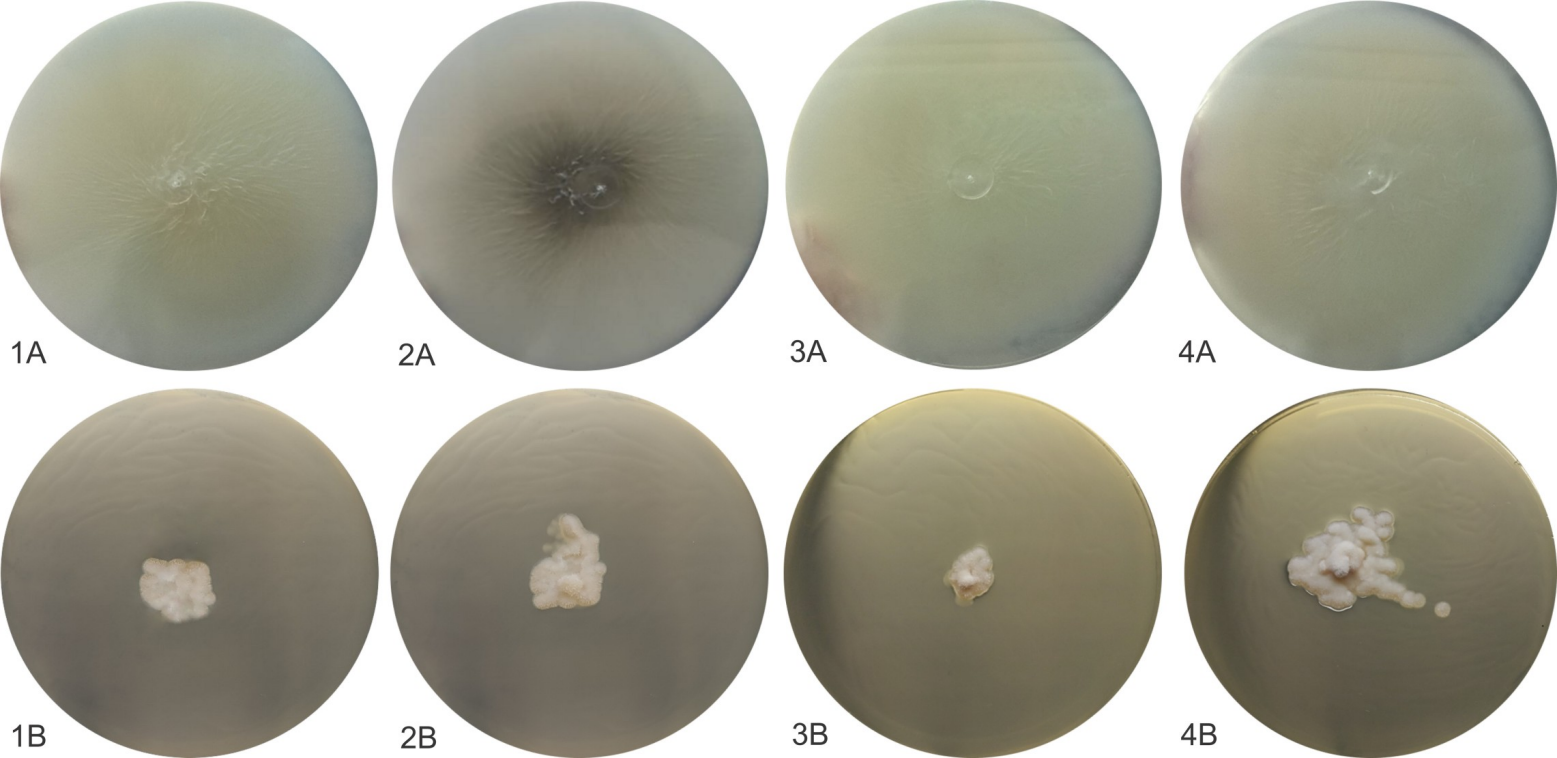
Macroscopic and microscopic morphologies of *S*. *chilensis* isolates. (A) Colony of *S*. *chilensis* developed on PDA at 30°C, (B) Colony of *S*. *chilensis* developed on PDA at 37°C.

**Fig 3:**
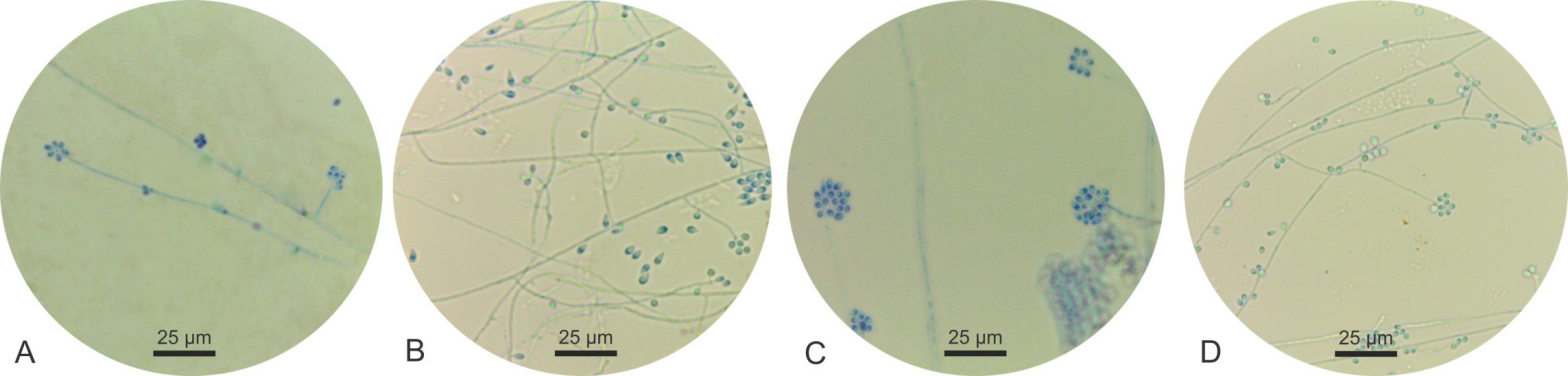
Microscopic morphologies of *S*. *chilensis* URM434 (A) URM435 (B), URM2439 (C), URM2865 (D).

All isolates identified at the species level by molecular screening as *S*. *schenckii sensu stricto* (URM 1013, URM 3686, URM 4080, URM 4252, URM 4291, URM 4861, URM 5111) and *S*. *chilensis* (URM 434, URM 435, URM 2439, URM 2865) were phenotypically characterized as reported in Table 2. However, the strains identified as *S*. *chilensis* received attention highlighted in this study and had images obtained of their colonies and micromorphological structures. After three weeks of incubation at 30°C, colonies on PDA were moist, glabrous to cotton-like surface, white to discreet dark brown (Fig 2: 1A-4A). Microscopically these fungi presented thin, septated hyaline hyphae. All isolates sporulated on CMA after 12 days at 30°C and developed intercalate or terminal conidial clusters on sympodially conidiophores. The conidia were hyaline or slightly pigmented, usually obovoid. These sessile conidia were globose, elongated (Fig 3A–3D).

**Table 2:** Phenotypic characteristics and genotypic of *Sporothrix* species deposited in Culture Collection of URM. x(presence), 0 (ausence)* However, microscopic examination determined that this strain produced dematiaceous triangular conidia, structures not previously described on this strain, which have been only associated previously with strains of *S*. *schenckii*.

ISOLATES	CONIDIA					ASSIMILATION TEST					POLYPHASIC TAXONOMY	
	DEMATIACEOUS				HYALINE	COLONY DIAMETER IN mm						
	Globose N	Obovoidal N	Triangular N	Elongated N	Hyaline N	30°C	37°C	Glucose	Sucrose	Raffinose	Phenotypic identification	Molecular identification
***URM434***	0	0	0	x	x	72.25	6	+	+	+	*Sporothrix* spp.	*S*.*chilensis*
***URM435***	x	x	0	x	x	63.5	6.75	+	+	+	*S*.*mexicana*	*S*.*chilensis*
***URM2439***	0	0	0	x	x	68.5	4.5	+	+	+	*Sporothrix* spp.	*S*.*chilensis*
***URM2865***	0	0	0	x	x	66,83	8,33	+	+	+	*Sporothrix* spp.	*S*.*chilensis*
***URM1013***	x	x	0	x	x	46,25	9	+	+	+	*S*.*schenckii*	*S*.*schenckii*
***URM3686***	0	x	x	x	x	52,75	13,25	+	+	+	*S*.*mexicana*	*S*.*schenckii*
***URM4252***	x	x	x	x	x	50.25	11.75	+	+	+	*S*.*mexicana*	*S*.*schenckii*
***URM4291***	0	x	x	x	x	53.25	9.75	+	+	+	*S*.*mexicana*	*S*.*schenckii*
***URM4861***	0	x	x	x	x	52.25	12	+	+	+	*S*.*mexicana*	*S*.*schenckii*
***URM5111***	0	x	x	x	x	52.75	10	+	+	+	*S*.*mexicana*	*S*.*schenckii*
***URM4080***	0	x	0	x	x	49	16	+	+	+	*S*.*schenckii*	*S*.*schenckii*
***CBS120339***	0	0	x	x	x	35	13	+	-	-	*S*. brasiliensis*	*S*.*brasiliensis*

### Growth rates and physiological analysis

The four isolates included in phenotypic study and the type strain of *S*. *brasiliensis* exhibited better growth at 30°C when compared to 37°C. At 37°C the isolates showed restricted growth, with 6 to 7 mm colony diameter after 21 days (Fig 2:1B-4B). When growth at 30°C was assessed, colonies achieved a mean diameter of 63.5 to 72.2 mm after 21 days. Carbohydrate assimilation tests revealed that all strains assimilated dextrose, and isolates available in this study (URM434, URM435, URM2439 and URM2865) were also able to assimilate raffinose and sucrose, differently of the *S*. *brasiliensis* type strain that was unable to assimilate raffinose and sucrose. Therefore, the *Sporothrix* isolates URM434, URM435, URM2439 and URM2865 demonstrated phenotypic characteristics of *S*. *mexicana* according to the previously established criteria in taxonomic key [3].

### *In vitro* antifungal susceptibility

For each antifungal susceptibility experiment, the inoculum control wells without antifungal drugs showed clearly detectable growth after the incubation period, indicating that all isolates were viable and that the conditions used were suitable for fungal growth.

The MIC values of amphotericin B, terbinafine and itraconazole against four *S*. *chilensis* isolates (URM434, URM435, URM2439 and URM2865) are shown in Table 3. These isolates showed good *in vitro* sensitivity to ITC (MIC range, 0.25–0.5 μg/mL) and TRB (0,125 μg/mL). However, moderate efficacy was observed for AmpB with MICs of 1–4 μg/mL.

**Table 3:** Minimal inhibitory concentrations (MIC) (μg/mL) of amphotericin B, itraconazole and terbinafine against *Sporothrix* sp. strains stocked in URM Culture Collection, UFPE, Recife, Brazil.

Culture Collection Access Number	Species	Antifungal–MIC^†^ values in μg.mL^-1^		
		AmpB^a^	ITC^b^	TRB^c^
^£^**URM 434**	*Sporothrix chilensis*	2	0.5	0.125
**URM 435**	*Sporothrix chilensis*	2	0.25	0.125
**URM 2439**	*Sporothrix chilensis*	4	0.5	0.125
**URM 2865**	*Sporothrix chilensis*	1	0.5	0.125
**URM 1013**	*Sporothrix schenckii*	1	0.5	0.125
**URM 3686**	*Sporothrix schenckii*	2	0.5	0.125
**URM 4080**	*Sporothrix schenckii*	0.125	0.125	0.125
**URM 4252**	*Sporothrix schenckii*	1	0.5	0.125
**URM 4291**	*Sporothrix schenckii*	0.5	0.125	0.125
**URM 4861**	*Sporothrix schenckii*	0.5	0.125	0.125
**URM 5111**	*Sporothrix schenckii*	2	0.5	0.125
^¥^**ATCC 22019**	*Candida parapsilosis*	0.25	0.125	0.125

^†^MIC—Minimal Inhibitory Concentration.

^a^AmpB- Anfotericin B

^b^ITC- Itraconazole

^c^TRB- Terbinafine

^£^URM- URM Culture Collection/UFPE/Brazil

^¥^ATCC- *American Typical Culture Collection*

Susceptibility profile similar was found in the seven *S*. *schenckii sensu stricto* isolates against ITC with MIC range of 0.125–0.5 μg/mL and TRB (0,125 μg/mL), however MIC values against AmpB displayed more susceptibility than *S*. *chilensis* with MIC range of 0.125–2 μg/mL as summarized in Table 3.

## Discussion

Sporothrichosis is endemic in Latin America [1] but its occurrence around the world has been massively reported [21]. The zoonotic transmission is related to the first epidemy of human sporotrichosis described in 1998, in the Rio de Janeiro, Brazil, with more prevalence of female patients engaged in domestic activities and persist at now [22]. Conventional route of transmission by traumatic inoculation with decaying vegetable and soil is also happening [2]. In the present study, the information about precise location of the body part of lesions are not available, although is possible associated these cases only the skin lesions as described in documents of deposit these isolates. Furthermore, herein was identified *Sporothrix* species in the Northeast Brazil, a poorly studied area in the field of sporotrichosis until the moment.

The historical Brazilian fungal service culture collection belonging to the Department of Mycology (URM) at Federal University of Pernambuco was established by Prof. Chaves Batista in 1954 and it is considered one of the most important Brazilian Fungal Culture Collection. Since the number and clinical impact of severe infections due to *Sporothrix* spp. has been increasing around Brazil, the identification of *Sporothrix* species with clinical relevance is one among several significant services now offered by the URM culture collection.

In this molecular screening seven isolates were identified as *S*. *schenckii sensu stricto* and four strains has been placed to be members of the *S*. *pallida* Complex corroborate with data of other recently study of Rodrigues and collaborators [10] but formed a distinct and well-supported clade, with *S*. *mexicana*, being nearest taxon with association as well as reported others studies [3, 9, 23].

Morphological and physiological features were did only four isolates identified by molecular screening as members of *S*. *pallida* Complex and this tests indicate the identity these isolates as *S*. *mexicana* although they presented slight variations when compared to the phenotypic markers proposed by Marimon and collaborators [3] which say that this species does not present growth at 30°C superior than 60mm in colony diameter. Our isolates presented between 63.5 and 72.5mm of colony diameter growth. However, Rodrigues and collaborators [10] also related growth above 60 mm in colony diameter in isolates identification as the new species *S*. *chilensis*. This new species is morphologically similar to *S*. *mexicana* and *S*. *pallida*, but their cultures and conidia does not produce large amount of dark pigment after extended incubation on PDA, SDA and CMA, as do *S*. *schenckii* and *S*. *mexicana* [3, 24]. Typical conidia (globose and obovoidal) observed in the genus *Sporothrix* were observed in isolates of *S*. *chilensis* alongside undifferentiated hyphae. However, secondary conidia present variation intra- and interspecifically on their shape and size of secondary conidia is however known to vary intra- and interspecifically corroborating with previously published data [3, 25, 13]. This description, from four isolates, helps to clarify the phenotypic characteristics of this rare species of the genus *Sporothrix*.

*Sporothrix* species identification has been based in a polyphasic approach by the combination of phenotypic and genetic techniques [8, 13, 23, 26]. In the description of the species *S*. *chilensis*, the authors suggested that single phylogenetic analyses of protein coding loci of just one gene provided support for the description of a new species and β-tubulin sequence data significantly confirmed *S*. *chilensis* as a distinct clade genetically close to *S*. *pallida*, *S*. *nivea*, *S*. *stylites*, *S*. *humicola*, and *Ophiostoma palmiculminatum* [10]. The use of β-tubulin sequencing as a single tool for *Sporothrix* sp species description is also supported by the study that established the *Sporothrix* complex, which states that this region is so descriptive as the CAL gene [27]. The phylogenetic analysis performed in this work has shown an evident segregation of *S*. *chilensis* from the other species that are included in the *S*. *schenckii* complex [2, 3, 6].

The isolation source of all hitherto published isolates of *S*. *chilensis* were either from natural environment or from a case of onychomycosis in Chile [10]. Our isolates were obtained from clinical skin samples in Brazil (Recife and São Paulo states), being the first report of this species in this country. Comparative genomic analyzes of DNA sequences grouped the fungi in the *S*. *pallida* clade as a distinct taxon paraphyletic to *S*. *mexicana* and identified as *S*. *chilensis*.

This report indicates that sporotrichosis may also be caused by *S*. *chilensis* in Brazil. In contrast, the isolates evaluated herein grew at 30°C lower than expected proposing that there may be significant intraespecific variation. Is important to highlight that this specie is not a known pathogen, presenting a mild-pathogenic potential and importance to immunosuppressed as previously described [10]. Considering that information of patients whose strains were obtained were not available, we are currently studying additional isolates to assess this hypothesis as well as studies additional will be necessary in these isolates obtained from endemic areas in the country for search more clinical cases.

According previously stablished epidemiological cutoffs values (ECV) for *Sporothrix* sp. pathogenic species [28], in our work, the obtained MICs for *S*. *schenkii senso strictu* to commercial drugs, used in sporotrichosis treatment, suggested that these strains are susceptible.

The study that stablishes these ECVs doesn’t provide these data for *S*. *chilensis* or other species of *S*. *pallida* complex. Nevertheless, in our work, the obtained MIC for itraconazole and terbinafine against *S*. *chilensis* are presenting in an ECV range corresponding to *S*. *brasiliensis* and *S*. *schenckii senso strictu* as previously provided [27], which suggests an applicable potential of these antifungals for therapy of sporotrichosis caused by this new species. On the other hand, amphotericin B may be not the best choice for the same treatment.

This was the first time that *S*. *chilensis* clinical isolates were tested against itraconazole, terbinafine and amphotericin B. Therefore, in order to provide more data regarding the resistance profile of this specie, more studies with a largest number of strains is demanded.

Here, we report a non-classic *Sporothrix* specie associated to clinical cases, *S*. *chilensis*, in patients from Brazil. This is a recently well-established species, and to our knowledge, this is the first description of *S*. *chilensis* isolation from skin of patients with sporotrichosis in our country indicating that this mycosis may also be caused by *S*. *chilensis* in Brazil.
